# Evaluation of the Microstructure and Properties of As-Cast Magnesium Alloys with 9% Al and 9% Zn Additions

**DOI:** 10.3390/ma18010010

**Published:** 2024-12-24

**Authors:** Lechosław Tuz, Vít Novák, František Tatíček

**Affiliations:** 1 Faculty of Materials Engineering and Industrial Computer Science, AGH University of Science and Technology in Krakow, 30-059 Krakow, Poland; 2 Faculty of Mechanical Engineering, Czech Technical University in Prague, 16629 Prague, Czech Republic; vit.novak@fs.cvut.cz (V.N.); frantisek.taticek@fs.cvut.cz (F.T.)

**Keywords:** magnesium alloys, mechanical properties, microstructure, weldability, forming, elevated temperature, thermal conductivity

## Abstract

The need to reduce energy consumption means that it is necessary to reduce the weight of vehicles. However, a thick wall of massive elements promotes the formation of casting defects, which must be removed by either plastic processing (straightening) or welding methods (surface and internal discontinuities). Basic alloys contain Al and Zn as the main alloying elements. The studies involved an evaluation of the microstructure and properties of alloys at ambient and elevated temperatures. The microstructure observation revealed a dendritic structure with the presence of low-melting eutectic, and intermetallic and Laves phases in the interdendritic areas. The presence of these phases may pose significant limitations during welding work. Thermal conductivity coefficient measurements showed that it is constant at temperatures up to 200 °C and is 49 W/mK for 9% Al and 77 W/mK for 9% Zn. The tensile test reveal that the most favorable tensile strength (120 MPa) occurs at temperatures of 150 °C for the 9% Zn alloy and at 180 °C for the 9% Al alloy.

## 1. Introduction

Magnesium alloys have been used in industry since the 1970s. Their use resulted from the high interest of the automotive and aviation industries in the production of parts with reduced mass, which translated into reduced fuel consumption. Nowadays, as a result of the development of electric cars, these materials are being reintroduced as the lightest construction materials. The favorable mechanical properties and general availability of alloys and their natural degradation mean that magnesium alloys as construction materials might see increasing application in both the production of massive and thin-walled elements and for work at elevated temperatures [1,2] and in medicine [3]. In production, sand casting techniques are used, especially in the case of massive elements, such as high-pressure die-casting technology for the production of thin-walled parts, [4] plastic processing [5,6,7], and machining [8]. In both cases, a significant limitation is high porosity, which on the one hand limits the mechanical and plastic properties of alloys and limits the use of heat treatment. On the other hand, the porosity and cracks of alloys located inside the walls can cause favorable properties in terms of work at elevated temperature due to the lack of increase in residual stresses. Porosity itself can be reduced by casting alloys under high-vacuum conditions. In this respect, there is a problem of the occurrence of low-melting structural components and phases, which, having a lower melting point than the matrix, crystallize last [9]. These limitations have urged research on the modification of the chemical composition of alloys by adding foreign particles to the matrix. Ganguly et al. [10] carried out research on reinforcing alloys with graphene nanoplatelets. He observed that the improvement in impression creep performance for as-cast nanocomposites is attributed to the reduced Mg_17_Al_12_ phase content, high dislocation density, and GNP strengthening. The studies performed indicate that the addition of an alloying element can significantly improve the mechanical properties and also creep resistance.

Kandemir S et al. [11] observed a gradual strengthening of the alloy and an improvement of mechanical properties by using carbon fiber of different lengths, but no improvement of the creep resistance was observed. Motavallian et al. [12] investigated the effect of solution treatment of the AZ91 alloy with regard to the microstructure, mechanical properties, and corrosion behavior of the friction stir back extruded AZ91/bioactive glass composite. The results obtained revealed that the presence of bioactive glass particles facilitates this re-precipitation process during friction stir back extrusion. Compared to the solid solutionized AZ91 alloy, the AZ91-3 vol% bioactive glass composite exhibits a 23% increase in the ultimate tensile strength (UTS), a 28% increase in the yield strength (YS), and a 30% decrease in the elongation. In turn, Cui et al. [13] studied the effect of the La/Nd ratio on the microstructure and tensile properties of AZ91-RE alloys. The results demonstrate that the alloy with a mass fraction ratio of La to Nd (wLa/wNd) of 3:2 exhibits the finest average grain size, the smallest mean NND, and the highest total number density of secondary phases. The results show that the formation of Al_11_La_3_ and Al_2_Nd exhibits significantly superior deformation resistance, shear deformation resistance, solid stiffness, and thermal stability compared to other secondary phases.

Studies have shown that similar effects can be obtained by adding Ti with different particles size [14], Ca [15,16,17], or nanotube and nanoplatelet [18]. The modification of the Mg_2_Si precipitates by Sb improves wear resistance [19]. Furthermore, such an improvement is closely related to the reduced susceptibility to microgalvanic corrosion, which is attributed to the reduced potential difference in voltage of Mg_2_Si relative to the Mg matrix, from 365 mV to 210 mV. The studies performed indicate that the presence of Ca in the alloy is beneficial both for mechanical properties and for corrosion and creep resistance [16,20,21]. The studies performed are important because they show that Ca, which is a natural impurity in magnesium alloys, should be treated as a beneficial alloying element. Corrosion resistance is an important utility feature of magnesium alloys and is related to both the microstructure and the chemical composition [20]. Among other particles, Ti-6Al-4V [22], NbB_2_ [23], Al_2_O_3_ [24], and SiCp [25,26] can also be used.

The tests performed show that during casting it is possible to obtain a favorable structure and mechanical and plastic properties, even at elevated temperatures. From the point of view of the manufacturing of parts, their joining and good weldability are also necessary. This is due to the need to join parts and perform repairs using welding methods [27]. The most commonly used welding methods include tungsten inert gas welding [28,29] and friction stir welding [30,31]. Welded joints are characterized by a structure different from the base metal [32], which requires heat treatment and machining [33,34,35]. Then, it is possible to obtain both favorable corrosion resistance and functional properties [36,37,38].

The article presents the results of structural and mechanical properties tests of magnesium alloys with the addition of 9% aluminum as reference material and 9% zinc as a new material. The addition of high amounts of Al or Zn make the structure prone to hot cracking due to the presence of low melted phases, and the cracking can be observed next to the weld as the effect of good thermal conductivity and low strength in elevated temperatures. To observe the change in the properties with increasing temperature, the analysis of the structure and mechanical properties in the as-cast state and under elevated temperature conditions were carried out. The alloys were tested in the state after casting into the mold because such material conditions are mostly in the repair work. The tests performed are important in the evaluation of the weldability of materials and are useful for the selection of technological conditions (i.e., preheating temperatures, interpass temperature, or post-welding heat treatment) for welding repairs of large castings, among others, for the aviation and automotive industries.

## 2. Materials and Methods

Two alloys cast by gravity into a sand mold of approximately 50 mm thick belonging to the AZ alloy group were used for the tests. In one alloy, the main alloying element was aluminum in the amount of approximately 9% and with a zinc amount of approximately 0.5%, which corresponds to the AZ91 alloy. The other was an alloy with approximately 9% Zn and 1% Al. The chemical composition of the alloys is presented in Table 1.

**Figure 1:**
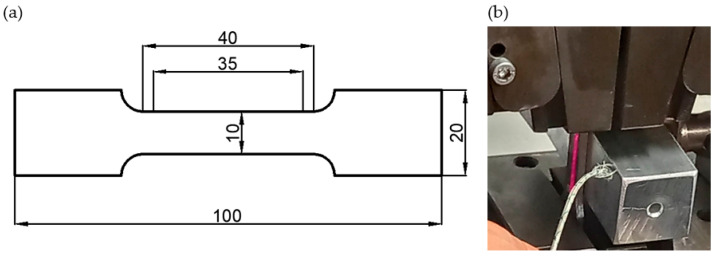
Sample for the tensile test (**a**) and the heating equipment (**b**), LabTest Model 5.100SP1, used for the tests.

The alloys in the as-cast state were not subjected to heat treatment, which made it possible to test the materials in the state immediately after casting, i.e., in the initial state for plastic working or welding processes. In the case of plastic working, it is crucial to obtain the appropriate plasticity, and in the case of welding processes, to limit the tendency for hot cracking. The test samples were mechanically cut from the plate from the area near the casting surface, approximately 10 mm thick, from the raw surface of the casting to avoid the porosity typical of massive castings. The samples for tensile tests were made by mechanical cutting. Tensile test samples with a measuring length of 35 mm and a width of 10 mm were cut using a water jet. The tests were carried out for two samples in each temperature. An illustration of the sample is shown in Figure 1a. Static tensile tests at ambient temperature and at elevated temperatures were carried out. Tests at elevated temperatures were carried out by contact heating of the samples using a temperature control system (Figure 1b). The heating element was heated inductively to a temperature 20 °C higher than the test temperature. The tests were carried out at temperatures of 120, 150, 180, and 240 °C (300 °C). The elongation was measured using a laser extensometer.

In order to determine the heating efficiency, measurements of the thermal conductivity of the alloys in the as-cast condition were carried out. The thermal conductivity coefficient was determined for temperatures ranging from 25 °C to 200 °C. The test procedure met the requirements of ASTM E1461 [39]. Thermal conductivity tests were performed on square samples with dimensions of 12.7 mm and a thickness of 10 mm.

Metallographic specimens were mounted in a thermosetting conductive resin, then ground on water-based abrasive paper, and polished using a polishing cloth and corundum suspension. Chemical etching was performed using a 2% alcoholic solution of nitric acid to reveal the structure.

Microstructure observations were performed on the etched specimens using light microscopy (LM) and scanning electron microscopy (SEM). Phase identification was performed based on the SEM-EDS method. Hardness measurements were made using the Vickers method with an indenter load of 10 kG (HV10).

## 3. Results

### 3.1. Microstructure Analysis

**Figure 2:**
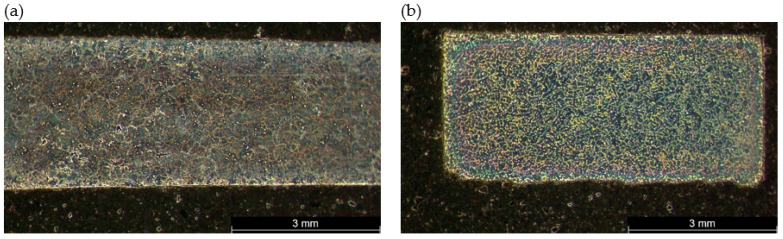
Macrostructure of the alloy in the cross-section: (**a**) Mg-Al-Zn, (**b**) Mg-Zn-Al.

**Figure 3:**
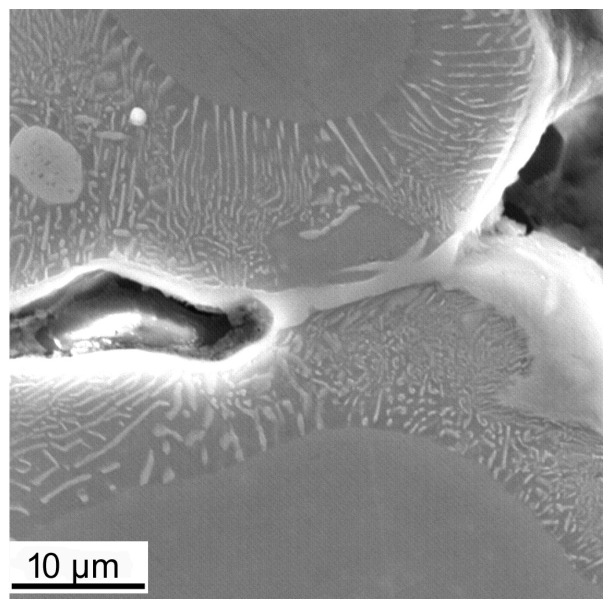
Microstructure near porosity in the Mg-Al-Zn alloy.

**Figure 4:**
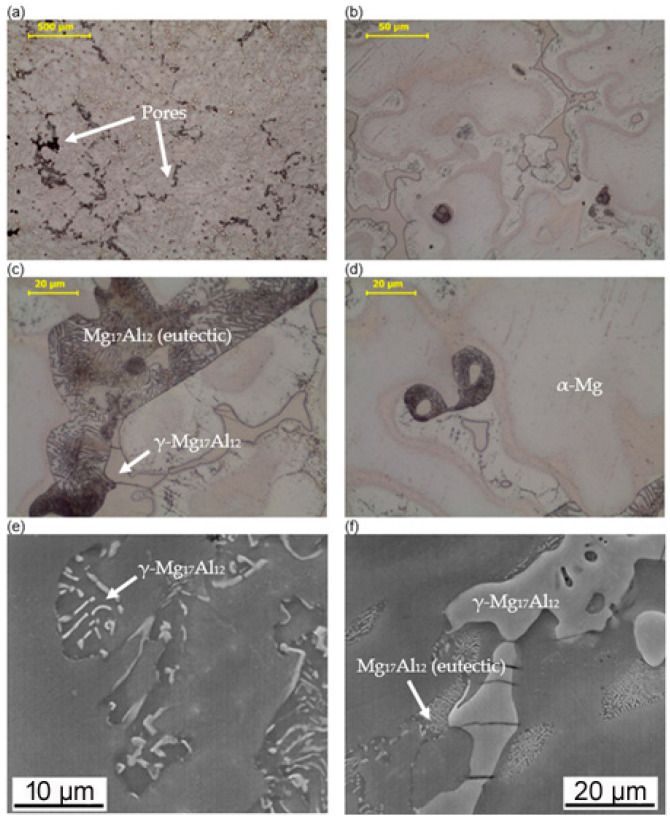
Microstructure of the Mg-Al-Zn alloy. (**a**–**d**) light microscopy, (**e**,**f**) SEM images.

**Figure 5:**
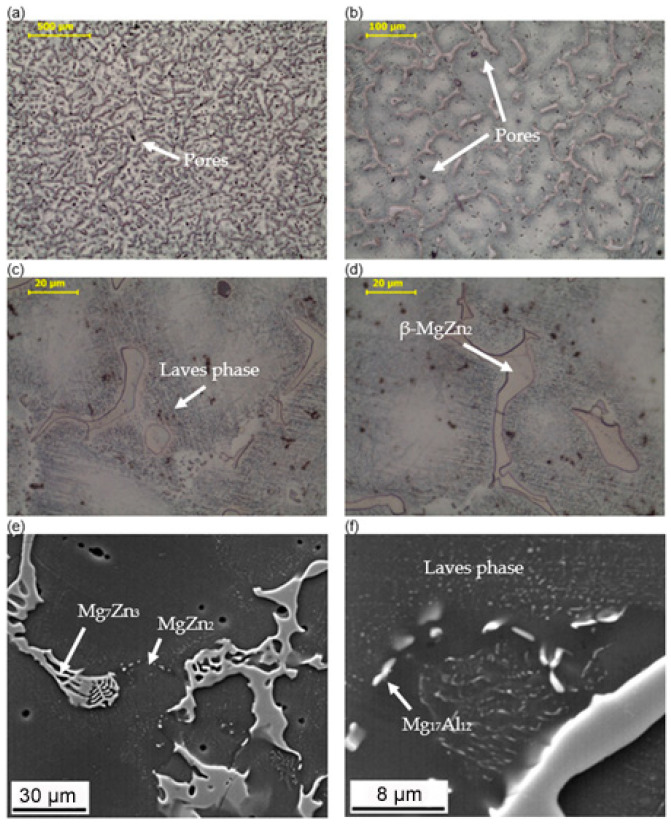
Microstructure of the Mg-Zn-Al alloy. (**a**–**d**) light microscopy, (**e**,**f**) SEM images.

**Figure 6:**
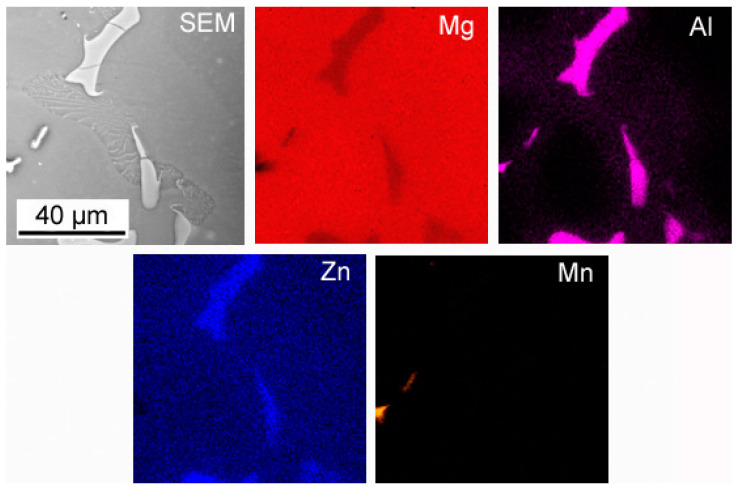
Map of the element distribution in the Mg-Al-Zn alloy.

**Figure 7:**
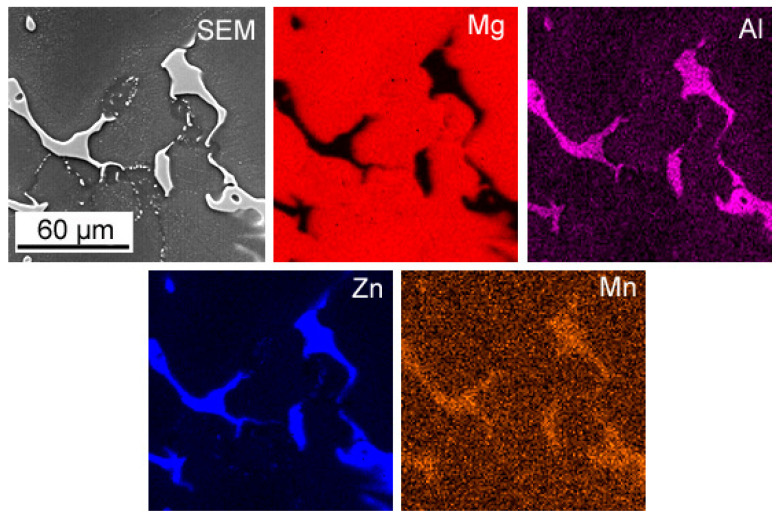
Map of the element distribution in the Mg-Zn-Al alloy.

The macroscopic examinations revealed that the Mg-Al-Zn alloy is characterized by local porosity that covers mainly interdendritic areas (Figure 2 and Figure 3). This indicates that during the crystallization of the alloy, there was not enough liquid to connect the dendritic areas. The exact location of the voids is observed in microscopic examinations (Figure 4a and Figure 5a). In both alloys, a dendritic structure was revealed, typical for cast materials, with precipitation of intermetallic and eutectic phases in the interdendritic areas (Figure 4 and Figure 5). The distribution maps of alloying elements revealed that alloying elements occur mainly in the interdendritic areas and are components that are characterized by the lowest melting temperature (crystallize last)—Figure 6 and Figure 7. The alloying elements that occur in the interdendritic areas form low-melting intermetallic phases. Based on quantitative EDS analysis (Table 2 and Table 3), the occurrence of the α-Mg phase in the dendrite cores of both alloys can be observed at individual points. In the Mg-Al-Zn alloy, the share of aluminum in the Mg-matrix is approximately 2.4% Al and 1.8% Zn. In the grain boundaries, the γ-Mg_17_Al_12_ phase is present mainly in the Mg-Al-Zn alloy, and probably the γ-Mg_17_Al_12_ and β-MgZn_2_ phases (Laves phase) or Mg_4_Zn_7_. The presence of impurities in the alloy, such as iron (Fe), copper (Cu), or nickel (Ni), probably creates intermetallic phases with zinc in both alloys. The low content of impurities causes these phases to be dispersive and indistinguishable in SEM-EDS. Nevertheless, they can adversely affect mechanical properties and corrosion resistance.

### 3.2. Thermal Conductivity Coefficient

**Figure 8:**
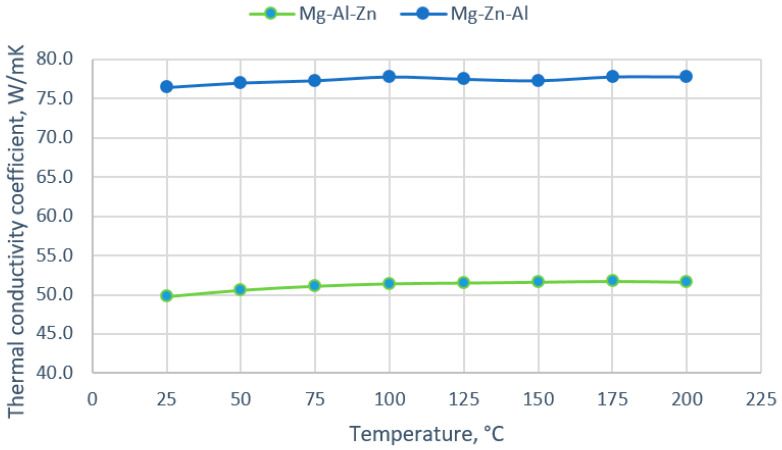
Change in the thermal conductivity coefficient in the temperature function.

Figure 8 shows the average values of the thermal conductivity coefficient depending on the temperature. It is visible that the value of the thermal conductivity coefficient shows a slight change in value. With the increase in temperature for the alloy with zinc and aluminum, the value increased by approximately 2 W/mK (Table 4). When the results of the changes in the thermal conductivity coefficient were analyzed, it could be observed that both the chemical composition and the porosity and nature of the structure affect the value of the coefficient. The alloy with the addition of aluminum has a greater number of pores and a greater precipitation of the γ phase at the grain boundaries; hence, it can be assumed that there is an effect of limiting the heat flow. On the other hand, the addition of zinc results in significantly more phases in the structure, mainly Laves phases, which can facilitate the flow of heat through the material.

### 3.3. Mechanical Properties

**Figure 9:**
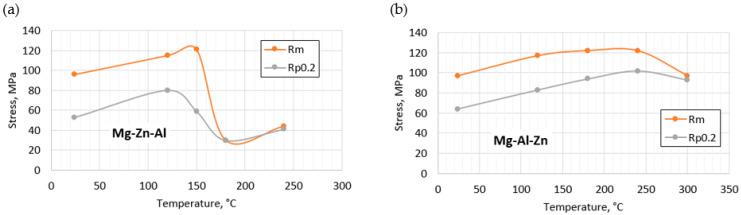
Change in mechanical properties in the function temperature of the (**a**) Mg-Al-Zn and (**b**) Mg-Zn-Al alloys.

The examination of the mechanical properties revealed that both alloys in the as-cast condition have relatively low plasticity. With increasing temperature, the plasticity of the alloy with the addition of zinc increases slightly (Table 5), while the addition of aluminum causes the plasticity of the alloy to decrease. The tensile strength (R_m_) in both alloys increased, reaching the highest value at 180 °C for the Mg-Al-Zn alloy (122 MPa), and in the case of the alloy with zinc, at 150 °C, it reached 121 MPa. In both cases, a similar highest tensile strength was obtained (Figure 9). The elongation of both alloys after casting was very low and reached the highest value of 1.15% for the Mg-Al-Zn alloy at a normal temperature and 0.48% at 150 °C for the Mg-Zn-Al alloy. Heat treatment (T5 or T6) is expected to improve the elongation.

**Figure 10:**
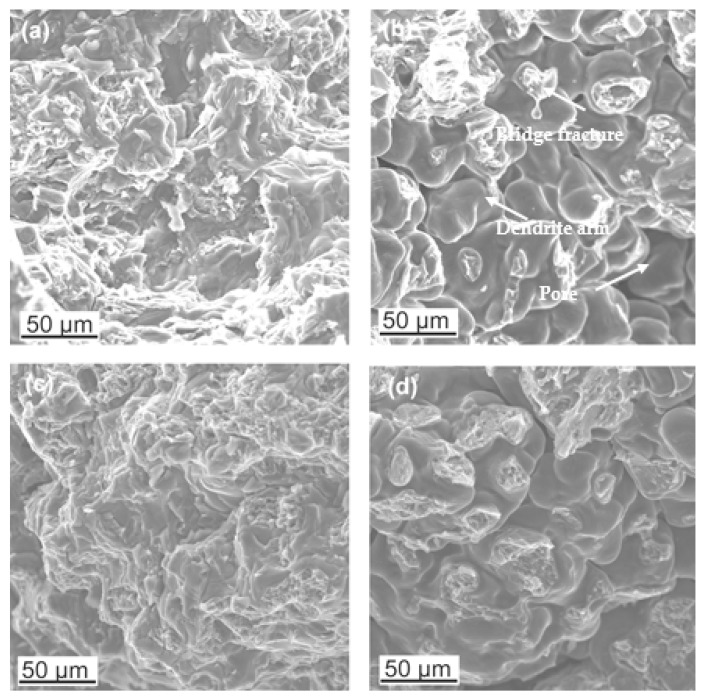
Fracture morphology of samples 92 (**a**,**b**) and 96 (**c**,**d**).

**Figure 11:**
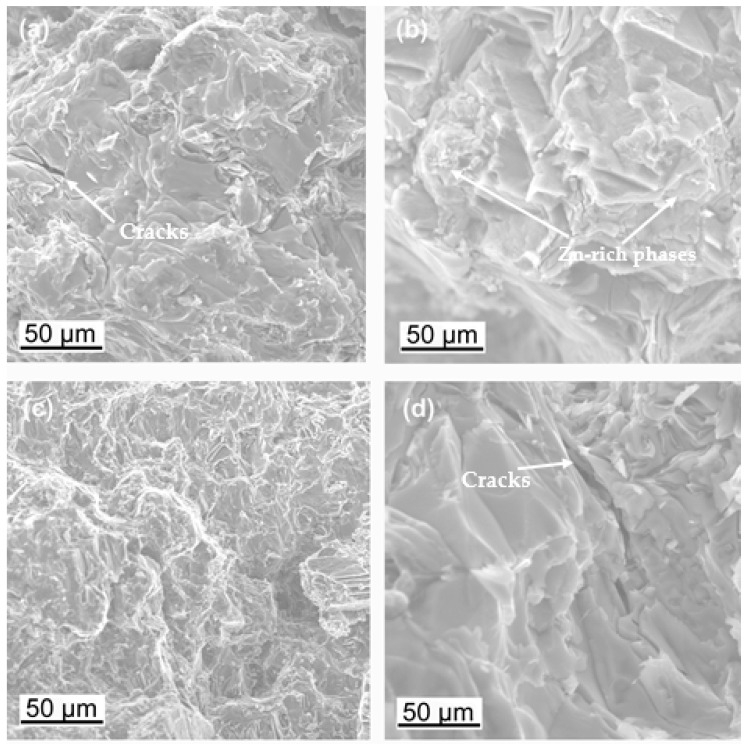
Fracture morphology of samples L2 (**a**,**b**) and L6 (**c**,**d**).

Fractographic studies of the fractures revealed that for the Mg alloy with aluminum, the strength was influenced by the porosity and shrinkage cavities were formed. The bridges formed between the dendrite arms were fractured or not connected at all (Figure 10). This causes the actual cross-sectional area of the samples to be smaller than that determined by the analysis of fracture stresses. The fracture observation of the alloy with zinc did not reveal any porosity. In both cases, the fracture observed was a brittle intergranular fracture. In the case of bridge fractures, a small lip was visible, indicating the flow of the material. Observations of the fractures of the zinc alloy revealed that there were numerous small transverse cracks on the surface, which indicates that the dispersive zinc-rich phases significantly increased the brittleness of the alloy (Figure 11).

The HV10 hardness measurements indicate that the Mg alloy with 9%Al has a high hardness of 83 HV10, while the alloy with zinc has almost half the hardness (46 HV10). This indicates that the Mg alloy with aluminum should be characterized by a tensile strength higher than that of the alloy with zinc. The results HV10 hardness measurements are presented in Table 6.

## 4. Discussion

The tests performed included the study of the mechanical properties and structure of magnesium alloys from the Mg-Al-Zn and Mg-Zn-Al groups, where the main alloying elements were aluminum and zinc in the amount of 9%, respectively. The alloys were tested as raw alloys after casting into a sand mold. The aim of the tests was to evaluate the possibility of conducting plastic processing or repair welding processes on the basis of the alloys’ properties. The alloys used in the tests are characterized by a dendritic structure where the dendrite cores consist of the Mg-matrix. In the interdendritic areas, alloying elements were located, forming intermetallic phases γ-Mg_17_Al_12_ and β-MgZn_2_ (Laves phases). These components crystallize last, and their presence can cause the heat input to the material during welding to induce their melting, although the rest of the material remains a solid. In the presence of a metallic liquid, welding stresses cause hot cracking. This effect was observed during the static tensile test, where the presence of porosity and shrinkage cavities caused only bridges between the arms of the dendrites with lower tensile strength. Thus, the alloy with the addition of 9% aluminum had a lower tensile strength than is typical for solid materials. The presence of intermetallic phases at the grain boundaries results in low plasticity in the alloys, and as a result, low susceptibility to cold plastic processing. This indicates that the materials used for forming should be heated to a temperature close to the melting point of the intermetallic and Laves phases.

Macroscopic studies have shown that the material at the surface is a solid material, and that porosity and shrinkage cavities occur within the material. This indicates that when thick-walled elements are cast into sand molds, the appropriate direction of material crystallization should be ensured. Then, all free spaces crystallize in the form of dendrites, and low-melting structural components fill the free spaces (interdendritic areas). Maintaining the mold at an elevated temperature is also necessary due to the presence of eutectic solidification in the interdendritic areas. This occurs between the dendrite and the precipitation of intermetallic phases. The nature of eutectic solidification causes the slight porosity of alloys to be the typical one for these alloys. Since the main component of eutectic solidification is aluminum, Mg-Al-Zn alloys are particularly susceptible to porosity and the appearance of shrinkage cavities. This is due to the fact that the dendrites have the structure of a solid solution of α-Mg, and the interdendritic areas are rich in intermetallic phase precipitates (Al-rich and Zn-rich). The occurring eutectic liquid, due to its lower melting point that is lower than the α-Mg solid solution, crystallizes as a mixture of crystals on the surfaces of dendrites due to the eutectic transformation. If the ends of the dendrite arms touch, then it is not possible for the eutectic liquid to flow into the free spaces and porosity is created. Since the alloy solidifies from the walls of the mold, if the possibility of gasses escaping from the mold is not ensured, the remaining gas is trapped between the dendrite cores (gas porosity). Zinc causes the formation of dispersive Laves phases, so the presence of Zn-rich eutectic is limited.

After casting, the alloys themselves are characterized by a relatively high tensile strength of approximately 120 MPa, which is maintained up to a temperature of 150–180 °C. Therefore, the use of preheating in this range to perform the welding process should limit cracking, as is shown in [40,41]. On the other hand, the alloys are characterized by a constant thermal conductivity coefficient in this range. Because of this, a significant part of the product is heated during heating. The temperature increase should also limit the occurrence of tensile stresses and thus the tendency to create internal discontinuities. Heating will be greater in the case of alloys with the addition of zinc due to the higher thermal conductivity coefficient of 78 W/mK, while the thermal conductivity of the Mg alloy with aluminum is only about 50 W/mK.

## 5. Conclusions

(1) Both alloy castings, with the addition of 9% Al and 9% Zn, have a dendritic structure, where the alloying components occur mainly in the interdendritic areas. In the interdendritic areas, mainly the γ-Mg_17_Al_12_ phase and the Laves β-MgZn_2_ phase occur. The presence of impurities in the alloy also promotes the formation of other intermetallic phases of a dispersive nature, which can be found both in the matrix and in the interdendritic areas. The presence of low-melting phases increases the hot-cracking susceptibility of both alloys. To avoid hot cracking it is necessary to use the preheating and low cooling rate. The preheating temperature should be in the range of the highest mechanical properties range.
(2) With increasing temperature to 200 °C, the thermal conductivity coefficient does not change significantly and is approximately 50 W/mK for the Mg-9Al-1Zn alloy and 77 W/mK for the Mg-9Zn-1Al alloy.
(3) With an increase in temperature, the initial tensile strength increases to 120 MPa at 150 °C for 9% Zn and 180–240 °C for 9% Al. A further increase in temperature causes a gradual decrease in the yield and tensile strength. The relatively low tensile strength of the Mg alloy with 9% Al is due to the presence of internal discontinuities in the material too.
(4) The Mg alloy with 9% Al is characterized by a higher hardness (83 HV10) in the as-cast state compared to the alloy with 9% Zn (46 HV10). The difference in hardness is the effect of the structure of the alloy, where aluminum, in addition to occurring in intermetallic and eutectic phases, also causes the strengthening of the matrix, and zinc forms finely dispersed precipitates.

The test conducted shows that both alloys are prone to hot cracking during welding. The hot-cracking susceptibility is the result of low-melting phases rich in Al or Zn presence in the structure, internal porosity, and good thermal conductivity. During welding, heat input is transferred, not only to the weld area but also to the base metal. This leads to cracking in the base metal. To observe and prove such phenomena, weldability tests of both materials are needed.

Massive castings after casting into sand molds are characterized by internal discontinuities resulting from gas entrapment (porosity) or the occurrence of low-melting eutectic and intermetallic phases. The presence of low-melting structural components leads to the formation of shrinkage cavities. The alloying component that promotes the formation of low-melting eutectics is aluminum.

## 6. Future Work

Detailed analysis of the precipitation in Mg-matrix and changes that occur in alloys under conditions of heat treatment and artificial aging. Weldability tests of both alloys to reveal the welding conditions the alloys are prone to, in order to avoid the hot cracking of as-cast alloys.

## Figures and Tables

**Table 1:** Chemical composition of cast alloys, %wt., Mg balance.

Alloy	Al	Zn	Mn	Fe
Mg-Al-Zn	8.62	0.54	0.21	0.36
Mg-Zn-Al	0.87	9.24	0.34	0.42

**Table 2:** Chemical composition of phases in the Mg-Al-Zn cast alloy; wt.%.

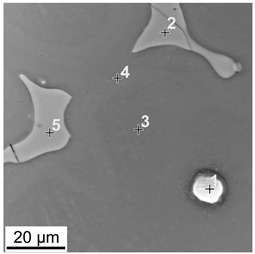	**No.**	**Zn**	**Al**	**Mn**
	1	0.7	32.8	50.3
	2	3.3	35.2	0.01
	3	0.3	2.9	0.05
	4	0.6	6.6	0.01
	5	0.8	36.7	0.02

**Table 3:** Chemical composition of phases in the Mg-Zn-Al cast alloy; wt.%.

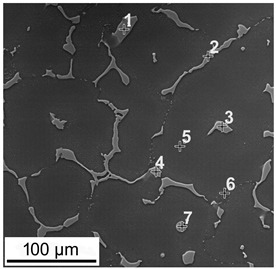	**No.**	**Zn**	**Al**	**Mn**
	1	64.1	13.4	0.02
	2	55.9	11.1	0.25
	3	63.1	8.8	0.08
	4	64.8	8.0	0.00
	5	5.4	1.3	0.05
	6	5.9	1.6	0.01
	7	47.3	4.9	0.03

**Table 4:** Cast alloys thermal conductivity coefficient measurements results, W/mK.

Temperature, °C	25	50	75	100	125	150	175	200
As-cast Mg-Al-Zn	49.8	50.6	51.1	51.4	51.5	51.6	51.7	51.6
As-cast Mg-Zn-Al	76.4	77.0	77.3	77.8	77.5	77.3	77.8	77.8

**Table 5:** Mechanical properties of tested alloys.

Temperature, °C	Mg-Zn-Al				Mg-Al-Zn			
	Sample No	R_p0.2_, MPa	R_m_, MPa	A, %	Sample No	R_p0.2_, MPa	R_m_, MPa	A, %
24	L2	53	96	0.20	92	64	97	1.15
120	L3	80	115	0.38	93	83	117	0.98
150	L6	59	121	0.48	94	94	122	0.94
180	L4	30	31	2.10	95	102	122	0.79
240	L5	41	44	1.67	96	93	97	0.38

**Table 6:** HV10 hardness measurement results.

Alloy	1	2	3	4	5	Average
Mg-Al-Zn	82	84	83	82	83	83
Mg-Zn-Al	44	46	46	47	45	46

## Data Availability

The original contributions presented in this study are included in the article. Further inquiries can be directed to the corresponding author.
